# Antioxidant and neuroprotective actions of resveratrol in cerebrovascular diseases

**DOI:** 10.3389/fphar.2022.948889

**Published:** 2022-09-05

**Authors:** Qing Wang, Qi Yu, Min Wu

**Affiliations:** ^1^ Shaanxi Prov Peoples Hospital, Shaanxi Prov Key Lab Infect and Immune Dis, Xian, China; ^2^ Shaanxi Key Laboratory of Ischemic Cardiovascular Diseases and Institute of Basic and Translational Medicine, Xi’an Medical University, Xi’an, China; ^3^ Department of Histology and Embryology, Xi’an Medical University, Xi’an, China; ^4^ Department of Pharmacology, College of Pharmacy, Shaanxi University of Chinese Medicine, Xianyang, China

**Keywords:** resveratrol, anti-oxidant, neuroprotection, stroke, vascular dementia

## Abstract

Cerebralvascular diseases are the most common high-mortality diseases worldwide. Despite its global prevalence, effective treatments and therapies need to be explored. Given that oxidative stress is an important risk factor involved with cerebral vascular diseases, natural antioxidants and its derivatives can be served as a promising therapeutic strategy. Resveratrol (3, 5, 4′-trihydroxystilbene) is a natural polyphenolic antioxidant found in grape skins, red wine, and berries. As a phytoalexin to protect against oxidative stress, resveratrol has therapeutic value in cerebrovascular diseases mainly by inhibiting excessive reactive oxygen species production, elevating antioxidant enzyme activity, and other antioxidant molecular mechanisms. This review aims to collect novel kinds of literature regarding the protective activities of resveratrol on cerebrovascular diseases, addressing the potential mechanisms underlying the antioxidative activities and mitochondrial protection of resveratrol. We also provide new insights into the chemistry, sources, and bioavailability of resveratrol.

## Introduction

Cerebrovascular disease (CVD) has become one of the most life-threatening diseases and represents the second leading cause of death and the main cause of serious disability, which predominantly clinically presents as acute neurological deficits (Ledbetter et al., 2021). CVD mainly includes fatal or non-fatal ischemic stroke, hemorrhagic stroke, transient ischemic attack, and vascular dementia (VaD). Due to vessel narrowing, thrombosis and emboli, these disorders may lead to reduced cerebral blood flow and even cerebrovascular rupture (Chowdhury et al., 2012). As the most common type of CVD, stroke is defined as an acute focal or global neurologic injury caused by the blockage of cerebral blood flow (ischemic stroke) and the sudden rupture of cerebral blood vessels (hemorrhage). Ischemic stroke accounts for approximately 80% of all strokes, and hemorrhagic stroke accounts for 20% (Strong, Mathers, and Bonita 2007). VaD is a cognitive disorder caused by impaired blood flow and vascular injury (Braun et al., 2019), which is the second most common type of dementia, being ranked behind Alzheimer’s disease. VaD accounts for 10–20% of all dementia, and the incidence of VaD among 65-year-old population has risen steadily over the years (Østergaard et al., 2016; Wolters and MA, 2019). Following changes in lifestyle and people getting older, the morbidity, mortality and disability rates of CVD have therefore risen sharply (Fu et al., 2021). However, there is still a lack of efficient therapy, particularly in medicine, protecting against oxidative stress.

**FIGURE 1:**
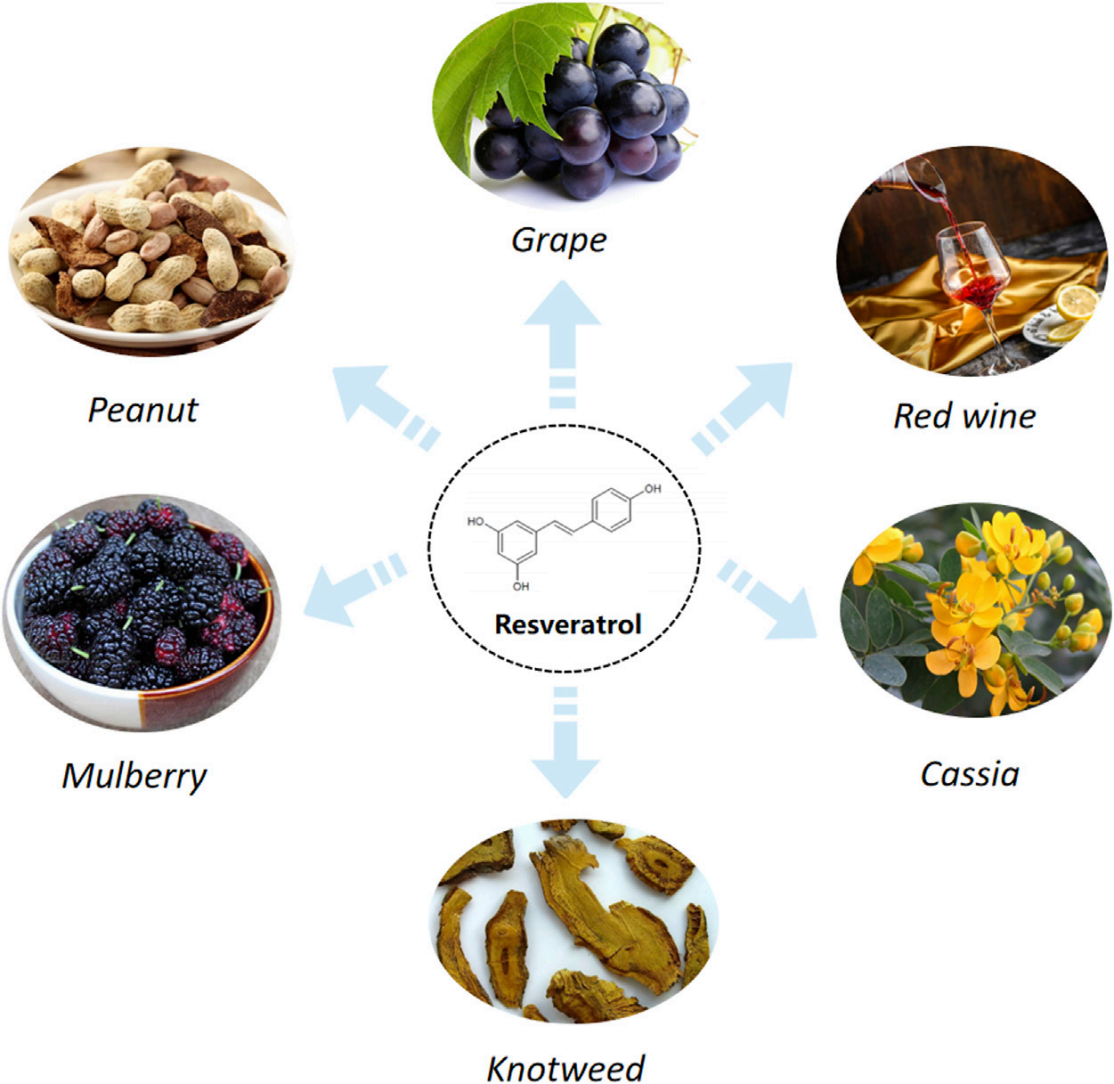
Resveratrol sources.

Recently, plant-derived natural antioxidants such as resveratrol (RES), quercetin, curcumin, and naringin are extensively used to treat and prevent various types of brain damage. It has been shown that RES has better antioxidant activity than other phyto-antioxidants (Khanduja and Bhardwaj 2003; Salehi et al., 2018). RES (3,5,40- trihydroxystilbene) is a natural polyphenolic compound, which is mainly extracted from grape skin/seed, vegetables, cereals, peanut skins, red wine, and tea (Yu, Fu, and Wang 2012) (Figure 1). Both *in vitro* and *in vivo* studies have shown that RES possesses diverse biological and pharmacological properties, which may be applied through antioxidant, anti-inflammatory, anti-apoptosis, anti-cancer, cardio-cerebrovascular protection (Shankar, Singh, and Srivastava 2007; Lopez, Dempsey, and Vemuganti 2015; Yang et al., 2015; Elshaer et al., 2018). It has been suggested that RES had cerebral-protective effects whereby it reduced radicals and upregulated the expression of antioxidant-related genes, including the endothelial nitric oxide synthase (eNOS) (Wang et al., 2011), superoxide dismutase (SOD) (Kovacic and Somanathan 2010), heme oxygenase-1 (HO-1) (Ren et al., 2011) and catalase (CAT) (Khan et al., 2013). Moreover, Sirtuin1 (SIRT1) is a nicotinamide adenine dinucleotide (NAD)-dependent deacetylase involved in both cellular stress and longevity (Horio et al., 2011). RES, as a chemical SIRT1 activator, can target the SIRT1 to maintain the homeostasis between prooxidants and antioxidants in mitochondria and exerts anti-inflammatory effects (Li et al., 2013). More evidence implicates that RES can attenuate various kinds of acute CVD, suggesting that the underlying mechanism is involved with its antioxidative effects (Ma et al., 2013; Hou et al., 2018). Here, we have summarized these novel works of literature to present the pharmacological role of RES in CVD, further assessing the anti-oxidative effect of RES and its potential mechanism.

## Structure and metabolism of RES

**FIGURE 2:**
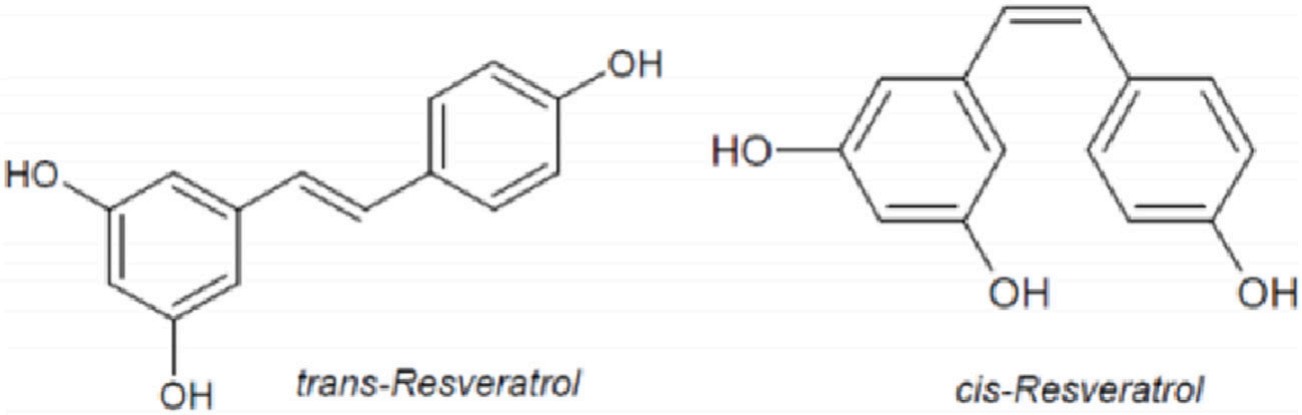
Chemical structures of trans-resveratrol and cis-resveratrol.

RES (C_14_H_12_O_3_; MW: 228.25 Da) is a natural antibacterial compound that binds to Flavin mononucleotide riboswitches and regulates gene expression (Sung and Dyck 2015). RES is a class of stilbene polyphenol molecules with dual structural isomeric forms: cis and trans (Figure 2). They possess comparable lipophilicity, but trans-resveratrol has a wider range of applications due to higher stability (Wallerath et al., 2002; Orallo 2006). Trans-resveratrol can be synthesized by pcoumaroyl coenzymes A (CoA) and malonyl CoA, whereas trans-resveratrol can also be converted into other cis-isomers after exposure to heat and ultraviolet irradiations (Shankar, Singh, and Srivastava 2007). RES is mainly transported through passive diffusion due to its small molecular weight and non-polar properties, but the oral bioavailability of RES is only 20% because of extensive metabolism in the gastrointestinal tract and poor intestinal absorption (Walle et al., 2004; Chen F et al., 2013). RES is quickly metabolized in the liver and binds to plasma albumin to promote drug uptake (Kuhnle et al., 2000). After oral and intravenous administration of RES, it has been shown that the main metabolites include sulfatesulfonylurea, 2-monosulfates, monofluorourea, 2-resveratrol monoglucuronides and dihydro resveratrol glucuronide (Calamini et al., 2010). In human urine, these metabolites were found in high concentrations (Boocock et al., 2007; Rotches-Ribalta et al., 2012). It is also reported that there was a ten folds increase in the half-life and plasma concentration of RES metabolites compared to native RES compounds in the human blood (Baur and Sinclair 2006). It suggested that even though its bioavailability was low and its metabolism and elimination were relatively rapid, RES had a relevant biological efficacy, which possibly was due to its conversion or interconversion into sulfonate and glucuronide metabolites (Vitaglione et al., 2005; Delmas et al., 2011). Besides, in animal studies, blood and serum levels of RES peaked 15 min after administration and then rapidly declined. Conversely, RES’s metabolites decreased rather slowly (Soleas et al., 2001). Above these results indicated that the bioavailability of RES metabolites was greater than that of RES (Goldberg, Yan, and Soleas 2003). It is reported that RES readily crossed the blood-brain barrier (BBB) and penetrated into brain tissue (Wang et al., 2002; Wang et al., 2004). They also demonstrated the levels of RES in the brain 4 h after intraperitoneal injection. A multicentric placebo-controlled phase II trial found that RES and its metabolites could penetrate the BBB based on the analysis of cerebrospinal fluid biomarkers (Turner et al., 2015). Besides, RES preserves BBB integrity. The study found that RES attenuated the BBB dysfunction by regulating the matrix metalloproteinase-9 (MMP-9) to limit the infiltration of leukocytes and other inflammatory mediators into the brain (Moussa et al., 2017). Nevertheless, there was no significant difference in the half-life times between the oral and intravenous administration. The half-life was 9.2 h after oral administration and 11.4 h after intravenous injection, and plasma concentrations of RES declined exponentially in a parallel of 72 h (Wang and Sang 2018).

On lipid peroxidation injury of brain synaptosomes, RES exerts the antioxidative potency. In this study, RES significantly scavenged the superoxide anion generated from rat forebrain mitochondria and inhibited ATPase activity with two EC50 values, 0.39 ± 0.15 nano-moles (nM) and 23.1 ± 6.4 micro-moles (μM) (Zini et al., 1999). Another study confirmed that the oxygen consumption of mitochondria was significantly inhibited by RES with a low EC50 of 18.34 picomole (pM) (Zini et al., 2002). Above these results showed that the RES had neuroprotection in CVDs via valuable anti-oxidant activities.

## Protective effects of RES on CVD

### RES and ischemic stroke

Ischemic stroke is caused by a reduction in cerebral blood flow, leading to energy dysmetabolism, neurological disorders, and cell death. Currently, the principal therapeutic treatment is to restore the cerebral blood flow whereby treatment with recombinant tissue plasminogen activator (rt-PA) destroys thrombi (clots), while rt-PA is also limited by the narrow therapeutic window (Warner et al., 2019). The main reason is that cerebral ischemia is a series of complicated pathological processes involving oxidative stress, inflammation, autophagy, mitochondrial dysfunction, and various signaling pathways (Tao et al., 2020). Chen et al. have revealed that RES extended the clinical therapeutic window of the rt-PA, suggesting that such an effect may be applied by reducing the MMP expression (Chen et al., 2016). Meanwhile, a study has demonstrated that RES preconditioning (RPC) provides a novel long-term window of cerebral ischemic tolerance for 2 weeks in mice (Khoury et al., 2019). These results provide evidence that RPC promotes a beneficial effect on cellular metabolisms such as glycolysis, mitochondrial respiration efficiency, elevated energy production (increased tricarboxylic acid cycle) as well as regulated oxidative phosphorylation and pyruvate uptake (Khoury et al., 2019). Numerous studies have shown that RES exerts neuroprotection in ischemic stroke by increasing antioxidant capacity as well as attenuating oxidative stress and inflammation (Al Dera 2017; Lei and Chen 2018). In such a process, SIRT1 is deemed as the primary molecular target of RES pharmacological actions (Borra, Smith, and Denu 2005; Della-Morte et al., 2009). In the cerebral ischemia-reperfusion-induced brain injury mice model, the mice were administered intraperitoneally with 30 mg/kg of RES and SIRT1/2 selective inhibitor before ischemia (Grewal, Singh, and Singh 2019). The results have shown that RES activates SIRT1, further promoting the improvement in neurobehavioral functions, the reduction in cerebral infarct volume, oxidative stress level, and acetylcholinestrase activity (Grewal, Singh, and Singh 2019). Other studies have also found that RES inhibited sulfonylurea receptor1 (SUR1) and aquaporin 4 (AQP4) expression by targeting specificity proteins transcription factors, thereby improving BBB damage, infarct area, cerebral edema and neurological deficit (Alquisiras-Burgos et al., 2020). In addition, Ma et al. have indicated that RES activated microglia and alleviated neuroinflammation via down-regulating the miR-155 expression (Ma et al., 2020). Even in the experimental stroke of aged female mice, the study shows that RES mitigates ischemic brain injury and inflammation (Jeong et al., 2016). The neuroprotective effects of RES on various ischemic brain injury models are shown in Tables 1, 2.

**TABLE 1:** Summary of the most relevant preclinical studies *in vivo* evaluating the effects of resveratrol administration on animals subjected to cerebrovascular diseases, including ischemic stroke, hemorrhage stroke, and vascular dementia.

Disease	Experimental model	Animal Sex/Age	Dose and duration of study	Outcome of study	References
Ischemic stroke	Occlusion of the middle cerebral artery 2 h by insertion of a silicone-coated 8-0 and the suture was removed to restore the blood flow	Male SD rats/age 8–12 weeks	RES30 mg/kg, intraperitoneally, for 7 days	RES reduced neurological deficit scores, promoted proliferation of neural stem cells, inhibited astrocyte and microglia activation by the Shh signaling pathway	Yu et al. (2021)
Ischemic stroke	Occlusion of the right common carotid artery (RCCA) and right middle cerebral artery for 60 min	Male SD rats/not mentioned	RES 20 mg/kg, intraperitoneally, for 10days	RES reduced levels of MDA, Ferrum (Fe), Copper (Cu), and Aluminum (Al) and increased the anti-oxidants enzyme SOD and CAT activity	Lin et al. (2021)
Ischemic stroke	Ligation of the right middle cerebral Artery (RMCA) and RCCA for 1 h	Male SD rats/not mentioned	RES20 mg/kg, intraperitoneally, for 10 days	RES increased trace element concentrations of Magnesium (Mg), Zinc (Zn), Selenium (Se), SOD and CAT antioxidant activity	Ro, Liu, and Lin (2021)
Ischemic stroke	Occlusion of the middle cerebral artery 2 h by insertion of a silicone-coated 8-0 and the suture was removed to restore the blood flow	Male Wistar rats/not mentioned	RES1.9 mg/kg, tail vein injection, at the onset of reperfusion	RES reduced the cerebral region damage and diminishes glucose transporter 3 (GLUT3) expression at the mRNA and protein level in astrocytes which might depend on adenosine 5‘-monophosphate (AMP)-activated protein kinase (AMPK) activation	Aguilar et al. (2020)
Ischemic stroke	Occlusion of middle cerebral artery 24 h	Male SD rats/Adult	RES 30 mg/kg, intraperitoneally	RES improved the neurological behavior, brain edema and brain infarction by upregulating the p-Akt and p-glycogensynthasekinase-3β (p-GSK-3β) expression levels	Park et al. (2019)
Ischemic stroke	Occlusion of the middle cerebral artery 2 h and the suture was removed to restore the blood flow for 24 h	Male Wistar rats/not mentioned	RES1.8 mg/kg, tail vein injection	RES decreased the infarct area,the production of superoxide anion, the overload of intracellular Ca^2+^ and increased the levels of phosphorylated AMPK.	Pineda-Ramírez et al. (2020)
Ischemic stroke	Right middle cerebral artery occlusion for 90 min and reperfusion immediately for 20days	Male SD rats/8 weeks	RES (10,100 mg/kg),intraperitoneally at 2 h on the onset of ischemia	RES significantly reduced the neurological deficit, cerebral infarct sizes, neuronal injury, decreased inflammation, and BBB disruption by downregulation of the toll-like receptor 4 (TLR4) pathway	Lei et al. (2019)
Ischemic stroke	Occlusion of the middle cerebral artery for 60 min, followed by reperfusion	Male C57BL/6 mice/8–10 weeks	RES 200 mg/kg, intraperitoneally for 3 days	RES attenuated systemic inflammation and neuroinflammation by modulating intestinal fora-mediated Th17/Tregs and Th1/Th2 polarity shift in SI-LP.	Dou et al. (2019)
Ischemic stroke	Occlusion of the middle cerebral artery 2 h and reperfusion immediately for 24 h	Male SD rats/Adult	RES 30 mg/kg, intraperitoneally, for 7 days	RES significantly decreased neuronal damage, and attenuated neuronal apoptosis via upregulating the PI3K/AKT/mTOR pathway by activating Janus kinase 2 (JAK2)/signal transducer and activator of transcription3(STAT3)	Hou et al. (2018)
Ischemic stroke	Occlusion of middle cerebral artery 24 h	Male SD rats/not mentioned	RES 100 mg/kg, intraperitoneally at 2 and 12 h after the onset of ischemia	RES significantly reduced the enzymatic activity of myeloperoxidase (MPO),suppressed the inflammatory factors, and upregulated the expression of cyclo-oxygen-ase 2(COX2) by activating PI3K/Akt pathway	Lei et al. (2019)
Ischemic stroke	Occlusion of the middle cerebral artery 2 h and reperfusion immediately	SD rats/not mentioned	RES 20 mg/kg, intraperitoneally for 7 days	RES alleviated cognitive impairment, downregulated inflammatory cytokines via modulating JAK/ERK/STAT pathway	Chang et al. (2018)
Ischemic stroke	Occlusion of the middle cerebral artery 60min and reperfusion immediately	Male SD rats/not mentioned	RES 100 mg/kg, intraperitoneally at the onset of reperfusion	RES attenuated inflammation, and upregulated autophagy by inhibiting NOD-like receptor protein 3 inflammasome (NLRP3) inflammasome activation through Sirt1-dependent autophagy activity	He et al. (2017)
Ischemic stroke	Occlusion of both carotid arteries for 30min	Male Wistar rats/Adult	RES 20 mg/kg, intraperitoneally, for30 days	RES exerted cerebral protection and inhibited inflammation by reducing interleukin-1β (IL-1β) and upregulating osteopontin	Al Dera (2017)
Ischemic stroke	Occlusion of the middle cerebral artery 90 min and reperfusion immediately for 24 h	Male Wistar rats/Adult	RES 20 mg/kg, orally, for 30 days	RES pre-administration reduced oxidative stress, inflammation, apoptosis, enhanced the levels of oxidized forms of DJ-1, and increased the Nrf2 expression via PI3K/Akt pathway activation	Abdel-Aleem et al. (2016)
Ischemic stroke	Occlusion of the middle cerebral artery 90 min and reperfusion immediately for 24 h	Male SD rats/not mentioned	RES 50 mg/kg, intraperitoneally, for 7 days	RES increased levels of IL-10, decreased tumor necrosis factor-α (TNF-α) and IL-6, increased frequencies of Tregs in the spleens and ischemic hemisphere, and improved the frequency and suppressive function of Tregs in the spleens	Yang et al. (2016)
Ischemic stroke	Occlusion of the middle cerebral artery 90 min and reperfusion immediately for 24 h	Male adult SD rats/2 months of age	RES 30 mg/kg, intraperitoneally at 1, 4, 6, 12, or 24 h before ischemia	RES before ischemia exerts a potent neuroprotective effect with an efficacious time window of at least 4 h via the national marine distributors association (NMDA) receptor-mediated ERK1/2-cAMP-response element binding protein (CREB) pathway	Li H et al. (2016)
Ischemic stroke	Occlusion of the middle cerebral artery 2 h and reperfusion immediately for 24 h	Male SD rats/7–8 weeks of age	RES 20 mg/kg, intraperitoneally, for 5 days	RES significantly reduced adenosine triphosphate (ATP) energy consumption and exerted neuroprotection by inhibiting PDEs and regulating the cyclic adenosine monophosphate (cAMP)/AMPK/SIRT1 pathway	Wan et al. (2016)
Ischemic stroke	Occlusion of the middle cerebral artery 90min and reperfusion immediately for 24 h	Male SD rats/Adult	RES 20 mg/kg, intraperitoneally at 0 and 20 h following reperfusion	RES prevented brain injury through ameliorating oxidative stress and reducing AQP4 expression	Li et al. (2015)
Ischemic stroke	Occlusion of the 4-vessel	Male Wistar rats/not mentioned	RES (1.10 mg/kg), intraperitoneally, for 21 day	RES attenuated doublecortin (DCX)/polysialylated-neural cell adhesion molecule (PSA-NCAM) expression, increased angiogenesis, improved spatial memory rete1ntion, and regulated corticosterone secretion	Girbovan et al. (2016)
Ischemic stroke	Occlusion of the 4-vessel	Male Wistar rats/not mentioned	RES (1.10 mg/kg), intraperitoneally, for 21day	RES exerted brain protection by increasing GLT-1 expression and inhibiting CD11b/c and glial fibrillary acidic protein (GFAP) expression	Girbovan and Plamondon (2015)
Ischemic stroke	Middle cerebral artery occlusion and reperfusion immediately for 24 h	Male SD rats/not mentioned	RES 50 mg/kg, intraperitoneally	RES attenuated the cerebral ischemia by maintaining the integrity of BBB via regulation of MMP-9 and tissue inhibitor of matrix metalloproteinases-1 (TIMP-1)	Wei et al. (2015)
Ischemic stroke	Middle cerebral artery Occlusion 30min and reperfusion immediately for 5.5 h	Male SD rats/not mentioned	RES (0.1 and 1 μM), Intracortical injection	RES exerted neuroprotection by activating either estrogen receptor subtype within the ischemic cortex of rats	Saleh, Connell, and Saleh. (2013)
Ischemic stroke	Middle cerebral artery occlusion 2 h and reperfusion immediately for 24 h	Male SD rats/not mentioned	RES 200 mg/kg, intraperitoneally, for 6 days	RES protected the brain through the Transient receptor potential channel 6/methyl ethyl ketone (TRPC6-MEK)-CREB and TRPC6-CaMKIV-CREB pathways	Lin et al. (2013)
Ischemic stroke	Middle cerebral artery occlusion for 90 min and reperfusion immediately	Male SD rats/8 weeks of age	RES 10 mg/kg, intravenously, for 20 days	RES ameliorated brain injury and attenuated neuronal apoptosis by downregulating the TGF-β-ERK pathway	Zhao et al. (2019)
Cerebral hemorrhage	Over-insertion of the intracranial internal carotid artery	Male SD rats/not mentioned	RES 100 mg/kg, intraperitoneally, 48 h prior to SAH	RES exerted neuroprotective effects, and prevented BBB disruption through the SIRT1/p53 signal pathway	Qian et al. (2017)
Cerebral hemorrhage	Fresh autologous arterial blood was slowly injected into the suprachiasmatic cistern for the 20s	Male SD rats/Adult	RES 60 mg/kg,intraperitoneally, 2 and 12 h post-SAH	RES provided neuroprotection, inhibited mitochondrial-dependent apoptosis, and improved mitochondrial biogenesis and antioxidative ability by activating the PGC-1α signaling pathway	Zhou et al. (2021)
Cerebral hemorrhage	Fresh autologous arterial blood was slowly injected into the suprachiasmatic cistern for the 20s	Male SD rats/not mentioned	RES (20,60) mg/kg, intraperitoneally, at 2 and 24 h after initial bleeding	RES attenuated neuronal apoptosis by the PI3K/Akt signaling	Zhou et al. (2014)
Cerebral hemorrhage	Over-insertion of the internal carotid artery	SD rats/not mentioned	RES 30 ml/kg, intraperitoneally, at 6 h after SAH	RES promoted functional brain recovery, prevented BBB disruption, and inhibited the activation of nuclear factor kappa-B(NF-κB) and downregulation of MMP-9 expression	Shao et al. (2014)
Vascular dementia	Induction of chronic cerebral hypoperfusion (CCH) induced by bilateral common carotid artery occlusion	Male Wistar/not mentioned rats/not mentioned	RES 40 mg/kg, intraperitoneally, for 4 weeks	RES effectively restored the synaptic plasticity and improved spatial memory via PKA-CREB activation	Li Z et al. (2016)
Vascular dementia	Occlusion of the 2-vessel	Male Wistar rats/3 months of age	RES 20 mg/kg,intraperitoneally, for 7 days	RES attenuated pyramidal cell death in the CA1 hippocampal subfield, prevented both spatial working and References memory impairments, and increased the nerve growth factor (NGF) levels	Anastácio et al. (2014)
Vascular dementia	Bilateral common carotid artery occlusion (BCCAO)	Male SD rats/2 months of age	RES 20 ml/kg, intraperitoneally, for 4 weeks	RES exhibited neuroprotective effects, and inhibited apoptosis and oxidative stress injury	Zhang et al. (2019)
Vascular dementia	BCCAO	Male SD rats/not mentioned	RES 50 mg/kg, intragastrically, for 9 weeks	RES improved cognitive function and reduced neuronal damage and neuronal apoptosis by activating autophagy and regulating the Akt/mTOR signaling pathway	Wang et al. (2019)

**TABLE 2:** Summary of the most relevant preclinical studies *in vitro* evaluating the effects of RES administration on animals subjected to CVD, including ischemic stroke, hemorrhage stroke and vascular dementia.

Disease	Experimental model	Dose and duration of study	Outcome of study	Reference
Ischemic stroke	Primary cortical neurons were subjected to oxygen-glucose deprivation/reperfusion *in vitro*	RES 5 μM	RES reduced neurological deficit scores, promoted proliferation of neural stem cells, inhibited astrocyte and microglia activation by the Shh signaling pathway	Yu et al. (2021)
Ischemic stroke	Primary cortical neurons were subjected to oxygen-glucose deprivation/reperfusion *in vitro*	RES 10 µM	RES improved cell viability and suppressed oxidative stress by stimulating the PTEN-PINK1/Parkin-mediated pathway	Ye, Wu, and Li (2021)
Ischemic stroke	SH-SY5Y cells were subjected to oxygen-glucose deprivation	RES 10 μM	RES rescued mitochondrial deficiency, increased the Bcl-2 and CREB expression, and inhibited caspase 3 and 9 activity via increasing expression of AMPK and p-AMPK	Lin et al. (2020)
Ischemic stroke	Primary rat cortical neurons were subjected to oxygen-glucose deprivation/reperfusion *in vitro*	RES 40 mM	RES treatment at different times increased neuronal viability, decreased the LDH and SOD activity, and inhibited neuronal apoptosis via enhancing the activation of the Nrf2 pathway	Yang et al. (2018)
Ischemic stroke	HT22 cells were subjected to oxygen-glucose deprivation/reperfusion *in vitro*	RES 10 µM	RES attenuated cytotoxicity, and oxidative stress and repaired DNA damage by upregulating APE1 activity and level	Jia et al. (2017)
Ischemic stroke	PC12 cells were subjected to oxygen-glucose for 6 h deprivation/reperfusion 24 h*in vitro*	RES 25 µM	RES significantly increased the cell viability and decreases ROS generation, intracellular Ca^2+^ levels, and hypoxia associated transcription factors	Yu, Fu, and Wang. (2012)
Cerebral hemorrhage	Primary cultured cortical neurons were stimulated with oxyhemoglobin (oxyHb) to induce SAH.	RES 20 µM	RES protected primary cortical neurons against oxyHb insults, including reducing the proportion of neuronal apoptosis, alleviating neuronal degeneration, and improved cell viabilities	Zhang X et al. (2017)

### RES and hemorrhage stroke

Cerebral hemorrhage is a cause of cerebral brain vasospasm (Souissi et al., 2010). More importantly, subarachnoid hemorrhage (SAH) results in long-term cognitive and neurological outcomes (Bederson et al., 2009; Suzuki 2015). Studies have revealed that oxidative damage and endoplasmic reticulum (ER) stress lead to the early stage of brain injury (Ayer and Zhang 2008; Nakka, Prakash-Babu, and Vemuganti 2016). In recent years, there has been a functional link between oxidative stress and ER stress (Ilieva et al., 2007; Zhang 2010). He et al. showed that oxidative stress increased the accumulation of reactive oxygen species (ROS) in the ER stress, and upregulated the expression of Glucose-regulated protein 78 (GRP78) (He et al., 2008). Zhang et al. also proposed that ROS can target ER-based calcium channels, leading to the release of calcium from the ER to the cytosol (Zhang 2010). Increased cytosolic calcium can form a positive feedback loop to produce more ROS. GRP78 is a molecular chaperone with important functions at the cellular level, including the regulation of intracellular calcium, protein folding, and ER stress (Chiang et al., 2011). As a monitor of ER stress, GRP78 is associated with oxidative stress and stabilization of calcium homeostasis (Kudo et al., 2008). It is reported that reduction of GRP78 expression inhibited the ER stress induced by oxidative stress (Pan et al., 2010). Xie et al. have revealed that RES ameliorated SAH-induced brain injury by attenuating oxidative damage, ER stress, and neuroinflammation (Xie et al., 2019). Regarded rats were treated with 60 mg/kg RES after SAH-induced brain injury. And they showed that it alleviated neurological deficits and brain edema, and reduced ROS and malondialdehyde (MDA) levels, indicating that the NF-E2-related factor 2 (Nrf2)/HO-1 pathway and suppressed GRP78 may involve with these effects (Xie et al., 2019). Another study demonstrated that RES significantly prevented the brain injury in hypertension-induced cerebral microhemorrhages, diminished hypertension-induced oxidative stress, and inhibited vascular MMP activation (Toth et al., 2015). Moreover, Post-SAH administration of RES attenuated neurological deficits, cerebral vasospasm, and microvessel thrombi through SIRT1-dependent activation (Diwan et al., 2021). A number of studies suggested that RES prevented BBB disruption, inhibited mitochondrial-dependent apoptosis, and was correlated with transforming growth factor-β-extracellular-signal regulated kinases (TGF-β-ERK), peroxisome proliferator-activated receptor-gamma coactivator (PGC)-1α, phosphatidylinositol three kinase/protein kinase B (PI3K/Akt) and et al. (Tables 1, 2).

### RES and vascular dementia

VaD is the second largest cause of dementia in the elderly and is mainly associated with cerebrovascular lesions as well as cognitive decline (Kovacic and Somanathan 2010). The primary pathological cause of VaD is the reduction of blood flow in cerebrovascular (Enciu et al., 2011). Chronic cerebral hypoperfusion is one of the most significant risk factors, which can lead to cerebrovascular degeneration, energy loss, oxidative stress, and inflammation (Jellinger 2007). Ma et al. showed that RES improved learning and memory ability in VaD rats. Specifically, RES exerted an anti-oxidative role via diminishing the MDA but increasing the SOD and glutathione (GSH) levels (Ma et al., 2013). Gocmez et al. established a streptozotocin-induced diabetic VaD rat model, and RES (20 mg/kg) was administrated intraperitoneally for 4 weeks, showing that RES prevented endothelial dysfunction, inflammation, and impairment of neurotrophin expression (Gocmez et al., 2019). Studies showed that a significant reduction of estrogens and progesterone was related to the onset of Alzheimer’s disease (Resnick and Henderson 2002; Jankowska 2017). Note, in the chronic cerebral hypoperfused and ovariectomized-female rats model, RES significantly alleviated brain injury by reducing astrocyte activation and its antioxidant and antiapoptotic effects. Above these findings may provide new insight into the potential clinical application in postmenopausal elderly women who suffer from VaD (Ozacmak, Sayan-Ozacmak, and Barut 2016). In addition, as shown in Table 1, RES regulates various proteins and signal pathways involved in the pathogenesis of VaD.

## Molecular mechanisms of RES on CVD

### Antioxidant actions of RES on CVD

A major mechanism of CVD may be ascribed to oxidative stress, which is mainly caused by the excessive accumulation of ROS and depleted activities of antioxidant enzymes (Orellana-Urzúa et al., 2020). Excess ROS includes superoxide anion, hydrogen peroxide (H_2_O_2_), and nitric oxide (NO), resulting in severe damage to cellular DNA, proteins, and lipids. It has been reported that the MDA and 4-hydroxynonenal were products of lipid peroxidation (Adibhatla, Hatcher, and Dempsey 2006). On the another hand, a variety of antioxidants such as SOD, CAT, GSH, and glutathione peroxidase (GSH-Pxs) protect from brain injury by eliminating ROS. Of note, the levels and activities of these antioxidants were markedly reduced in the animal model of CVD (Bharti and Garg 2019). Therefore, an increase in antioxidant defenses is an important strategy for blunting oxidative damage. Studies have shown that RES directly attenuates oxidative stress by inhibiting lipid peroxidation and upregulating the SOD and GSH activity in brain injury (Ma et al., 2013; Toth et al., 2015; Ozacmak, Sayan-Ozacmak, and Barut 2016; Zhao et al., 2019). Given that extensive ROS inhibits the generation of endothelial NOS (eNOS) and causes vascular endothelial damage, RES can prevent endothelial dysfunction by reducing oxidative stress and inflammation (Gocmez et al., 2019).

In addition, RES regulates trace elements to avoid ROS-induced oxidative damage. A variety of trace elements are important for maintaining normal brain functions. It is reported that Mg depressed oxidative stress and stabilizes the cell membrane (de Baaij, Hoenderop, and Bindels 2012). Zn is a part of the SOD structure and involves antioxidant production (Fang et al., 2013). Se has various beneficial effects, including protecting neurons, increasing antioxidant enzyme activity, and repairing DNA damage (Van Eersel et al., 2010; Song et al., 2014). Besides, increased Fe and Cu levels produce toxic hydroxyl radicals, leading to deleterious lipid peroxidation and oxidative injury (Allen et al., 2008; Murphy and Oudit 2010). Al and As are also considered to induce oxidative stress (Good et al., 1992; Miller et al., 2002; Cohen et al., 2006; Jomova and Valko 2011; Jaishankar et al., 2014). In a recent report, researchers have discovered that RES diminished the overload of Fe, Cu, As and Al, but increased Mg, Zn and Se levels to exert antioxidant effects in cerebral ischemic injury (Lin et al., 2021; Ro, Liu, and Lin 2021).

Nrf2 is a molecular target of oxidative stress through regulating abundant antioxidant enzymes and defense proteins (Vomund et al., 2017). Under normal physiological conditions, Nrf2 forms a Kelch-like ECH-associated protein l (Keap1)-Nrf2 complex in the cytoplasm. After stressful or inflammatory responses, Nrf2 is released from the Keap1/Nrf2 complex and translocates into the nucleus to combine with the antioxidant response element (ARE), initiating the transcription of downstream target genes (Kobayashi et al., 2004; Hayes et al., 2010). Many target genes are involved in the regulation of cellular redox homeostasis, such as NAD(P)H quinone oxidoreductase 1 (NQO1), HO-1, glutathione-s-transferase (GST) (Kumar and Mittal 2017). A recent study suggested that RES inhibited oxidative stress by upregulating the Nrf2 and HO-1 mRNA, thus protecting brain function (Yang et al., 2018).

Noteworthy, DJ-1 is activated during oxidative stress, which is therefore deemed as a crucial antioxidant regulator in brain science (Taira et al., 2004). Indeed, DJ-1 is an important modulator of Nrf2 and negatively regulates PI3K/Akt signal pathway (McNally et al., 2011). A number of studies have revealed that PI3K/Akt activation is apparently associated with antioxidative stress and anti-apoptosis (Liu et al., 2017; Yang et al., 2020). In contrast, PI3K activity is negatively regulated by phosphatase and tensin homolog deleted on chromosome ten (PTEN) (Almazari et al., 2012). In the central nervous system, PTEN has been considered a mediator of ROS production and cell apoptosis (Shang et al., 2022). At present, some studies have reported that DJ-1 activates the PI3K/Akt signal pathway by inhibiting PTEN to avert damage due to oxidative stress (Kim et al., 2005; Kim et al., 2009). In another study, RES reduced ROS generation and enhanced antioxidant enzyme activity. This effect is clearly linked to the reduction in oxidized levels of DJ-1, inhibiting PTEN activity and PI3K/Akt survival pathway activation (Abdel-Aleem et al., 2016).

PGC-1α is a transcription co-activator involved in mitochondrial biogenesis and function (Wareski et al., 2009). In addition, increasing evidence suggests that PGC-1α upregulates a serious of oxidative stress protective genes such as manganese SOD (Mn-SOD) to remove the ROS production (Valle et al., 2005; Orallo 2006). This remarkable antioxidant mechanism was also observed in the protective role of RES in the SAH model. In this study, RES increased PGC-1α expression and promoted PGC-1α nuclear translocation. Moreover, RES can apply to scavenge excess ROS, increasing the activity of SOD and improving the mitochondrial function and ATP levels to prevent neurological impairment after SAH. These results indicate that RES promotes mitochondrial biogenesis and decreases oxidative stress by activation of the PGC-1α signaling pathway in SAH (Zhou et al., 2021).

**FIGURE 3:**
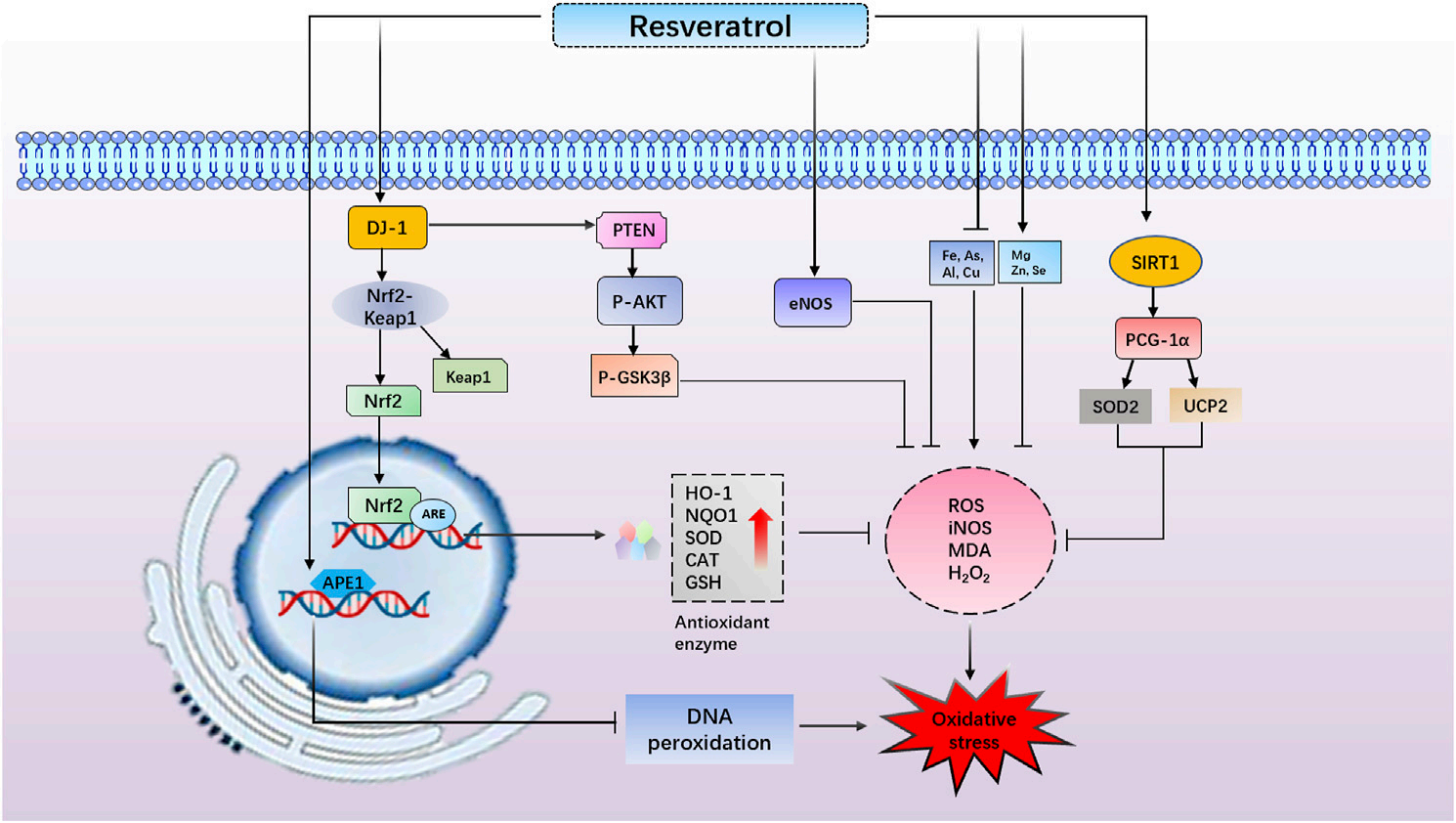
Resveratrol (RES) reduces apurinic/apyrimidinic endonuclease 1–induced oxidative DNA damage. RES increases DJ-1 protein expression, promotes NF-E2-related factor 2 (Nrf2) dissociating from Kelch-like ECH- associated protein l and induces Nrf2/antioxidant response element–dependent antioxidant enzyme (heme oxygenase-1 and NAD(P)H quinone oxidoreductase 1) transcriptions, thus exerting antioxidant effects. RES reduces reactive oxygen species generation and enhances antioxidant enzyme activity through reduction in oxidized levels of DJ-1, inhibiting phosphatase and tensin homolog deleted on chromosome ten activity and phosphatidylinositol 3 kinase/protein kinase B survival pathway activation. RES increases the generation of endothelial nitric oxide synthase to prevent oxidative stress. RES diminishes the overload of Fe, Cu, As, and Al but increases Mg, Zn, and Se levels to exert an antioxidant role. RES activates peroxisome proliferator–activated receptor–gamma coactivator–1alpha and increases superoxide dismutase 2 and UCP2 levels to promote mitochondrial biogenesis and thus decreases oxidative stress.

Oxidative stress commonly causes DNA peroxidation, which gives rise to DNA damage (Chen et al., 1997; Lan et al., 2003; Chen et al., 2011). APE1 is a ubiquitous and multifunctional DNA repair enzyme and participates in protein redox regulation (Dyrkheeva, Lebedeva, and Lavrik 2016; Laev, Salakhutdinov, and Lavrik 2017). Moreover, regulation of APE1 in neurons protects against ischemia-induced neuronal death (Ludwig et al., 1998; Vasko, Guo, and Kelley 2005). It has been reported that endogenous upregulation of APE1 reduced white matter infarct, promoting neurologic functional recovery after stroke (Stetler et al., 2016). Leak et al. have also revealed that the upregulation of APE1, either endogenously or through transgene overexpression, reduces oxidative DNA damage and protects neurons against acute ischemic damage (Leak et al., 2015). Consistent with prior studies, Jia et al. have found that RES obviously elevated the activity and level of APE1 (Jia et al., 2017). On the other hand, previous research has shown that strand breaks of apurinic/apyrimidinic sites are considered a marker of oxidative DNA damage, and 8-OHdG is also the specific product of DNA oxidative damage (Chen et al., 1997; Lan et al., 2003). Notedly, this study also demonstrated that RES reduced the level of 8-OHdG and AP sites in OGD/R-induced cell damage. In addition, the knockdown of APE1 blocked the neuroprotective and antioxidant effects of RES. The above results further showed that RES exerted a neuroprotective role in ischemic stroke, which was related to the APE1-induced oxidative DNA damage reduction and antioxidant defense system enhancement (Jia et al., 2017) (Figure 3).

### Mitochondrial protective effects of RES on CVD

Mitochondria are the major organelles involved in the synthesis of ATP in mammalian cells, which are essential for maintaining cellular homeostasis and function (Papa et al., 2012; Chaban, Boekema, and Dudkina 2014). Mitochondrial dysfunction plays an important role in the pathogenesis of CVD (Correia et al., 2010; Li et al., 2012; Chen et al., 2020; Han et al., 2020). Mitochondrial biogenesis is the process responsible for the synthesis of new mitochondria, which is the main component of the mitochondrial mass control system. Mammalian mitochondrial biogenesis and function require the coordinated action of both nuclear and mitochondrial genomes (Scarpulla 2008; Jornayvaz and Shulman 2010; Ungvari et al., 2011). PGC-1α regulates mitochondrial biogenesis by activating the nuclear respiratory factors 1 and 2 (NRF-1 and NRF-2) and increases the expression of mitochondrial proteins encoded by nuclear DNA (Virbasius, Virbasius, and Scarpulla 1993; Schreiber et al., 2004; Anderson and Prolla 2009; Scarpulla 2011). Among these proteins, Transcription factor A mitochondrial (TFAM) is an essential mitochondria transcriptor playing both a transcription and replication role in mitochondrial DNA (mtDNA) (Canugovi et al., 2010). Additionally, TFAM has been viewed as an important modulator of mtDNA homeostasis and repair (Picca and Lezza 2015; Filograna et al., 2021). The D-loop (mitochondrial displacement loop) was an mtDNA noncoding region, and it was the major control region for the regulation of mitochondrial genome replication and expression. The expression of the D-loop was used in evaluating mtDNA copy number and mitochondrial biogenesis (Shuwen, Xi, and Yuefen 2017). Recent evidence implicates the increasing mitochondrial biogenesis is thought to prevent mitochondrial dysfunction as well as attenuate CVD progression (Yin et al., 2008; Chen et al., 2010). Lin et al. have evidenced that RES rescued SH-SY5Y cells from OGD-mediated mitochondrial lower D-loop level and mitochondrial mass dimension. RES also rescued the transcript expression levels of PGC1-α, and mitochondrial genes (NRF-1 and TFAM) in OGD-treated SH-SY5Y cells (Lin et al., 2020). Besides, AMPK activity is important for maintaining mitochondrial biogenesis homeostasis (Zhang J et al., 2017). These studies have shown that activation of AMPK protected neurons against mitochondrial dysfunction, and oxidative stress, further suggesting that AMPK may be critically important in preventing neuronal cell damage (Zhang J et al., 2017; Nanjaiah and Vallikannan 2019). Lin et al. also have found that RES improved the expression of AMPK and p-AMPK in OGD-exposed SH-SY5Y cells. It implies that RES reverses OGD-induced SH-SY5Y cell damage caused by mitochondrial dysfunction in an AMPK-dependant manner (Lin et al., 2020).

Mitochondria also have crucial functions in a number of essential cellular processes, such as the regulation of ROS production, tricarboxylic acid cycle (TAC) and respiratory chain regulation (Madamanchi and Runge 2007; Peng et al., 2019). In the brain, oxidative damage decreases enzyme activity of the respiratory chain, resulting in mitochondrial dysfunction (Szeto 2008; Huang et al., 2013; Han et al., 2020). The respiratory chain dysfunction promotes electrons leakage and excess ROS production (Murphy 2009). ROS forms a positive feedback loop, which can cause damage to the inner membrane integrity of the mitochondrial, mitochondrial depolarization, and the opening of mitochondrial permeability transition pore (MPTP) (Szeto 2008). ROS mainly consists of H_2_O_2_ and superoxide anion radical. H_2_O_2_ and superoxide anion radical lead to alterations in the function of the respiratory chain (Turrens 2003). There are several antioxidant defense mechanisms in mitochondria, including enzyme and non-enzymatic defenses. Among the enzymatic antioxidant defenses, Mn-SOD takes a crucial role in the converting superoxide anion radical into H_2_O_2_ through reacting with glutathione peroxidase (GPx) or CAT, generating water (Flynn and Melov 2013; Lu 2013; Morris et al., 2014; Barrera et al., 2016). Reduced GSH is the main nonenzymatic antioxidant defense (Lu 2013). GSH is produced in the cytosol and is necessary to attenuate redox impairment through the reactions of Mn-SOD (Flynn and Melov 2013) and from mitochondrial complexes (Zorov, Juhaszova, and Sollott 2014). Therefore, protective intervention in the mitochondrial function might contribute to the amelioration of ischemia-related brain damage. Studies have shown that RES and mitochondrial function have also been linked to ischemia-reperfusion injury (Zini et al., 1999; Kipp and Ramirez 2001; Zini et al., 2002). The RES can preserve the function of brain mitochondria by reducing the generation of ROS in the mitochondria after hypoxia-reoxygenation (Morin et al., 2003). The mitochondrial respiratory enzyme COX was also examined to evaluate the mitochondrial function (Ortiz-Ruiz et al., 2021). In the present study, it is very likely that RES significantly rescues SH-SY5Y cells from OGD-mediated mitochondrial deficiency (maximal respiratory function, ATP content, COX activity, and mitochondrial membrane potential) (Yousuf et al., 2009; Lin et al., 2020). Remarkably, Khoury et al. reported that RPC promoted an increase in glycolysis and mitochondrial respiration efficiency. Additionally, genes involved in pyruvate uptake, TAC cycle, and oxidative phosphorylation were more highly expressed, indicating protection on mitochondrial energy-producing pathways during cerebral ischemia (Khoury et al., 2019). For the treatment of SAH, RES resulted in the activation of Sirt5. The activation restored mitochondrial metabolism dysfunction and alleviated early brain injury as well as desuccinylation of citrate synthase and ATP synthase (Xiao et al., 2021). There is also evidence that in the VaD model, RES significantly reduces mitochondrial ROS generation, lipid peroxidation, and protein carbonyls. Beyond this, significant improvements in redox ratio and Mn-SOD activity were observed with RES (Yadav et al., 2018).

## The adverse reactions and clinical efficacy of RES

Although RES intake has potent pleiotropic effects in humans and is well tolerated (Ramírez-Garza et al., 2018), others have reported toxic effects of RES *in vitro* and *in vivo* (Calabrese, Mattson, and Calabrese 2010; Cottart et al., 2010; Salehi et al., 2018). It is known that RES has beneficial antioxidant activity, but it appears that RES intake has negative effects on metabolic function, endothelial health, inflammation, and cardiovascular markers in human patients (Ramírez-Garza et al., 2018). In one study, healthy volunteers were given RES doses of 25 mg, 50 mg, 100 mg, and 150 mg at intervals of 4 hours for 48 h. Finally, in some participants, mild adverse effects were reported, such as headaches, dizziness, and epididymitis (Almeida et al., 2009). In obese postmenopausal women, administration of 1 g RES for 12 weeks resulted in negative effects. The liver enzyme levels of one of the 34 subjects were elevated. In addition, 30% of the subjects experienced diarrhea, and 27% had increased total cholesterol (Chow et al., 2014). It is reported that a 450 mg/day dose of RES was a safe dose for a 60 kg person (Moon et al., 2004). RES (1,000 mg/day) was found to inhibit cytochrome P450 isoenzymes such as CYP1A2 and CYP2C9, resulting in interactions with many other drugs (Detampel et al., 2012). In most cases, these interactions could diminish the activity of these drugs. It indicates that RES administration (over 1000 mg/day) causes differences in the pharmacokinetics of drugs administered concurrently (Detampel et al., 2012). Moreover, intracellular redox imbalance in endothelial cells can lead to endothelial dysfunction, which is an important step in the progression of CVD (Cai and Harrison 2000). Numerous studies have demonstrated RES provided protection via its antioxidant impact on the endothelium (Cao and Li 2004; Chen M et al., 2013). However, RES has been shown to increase intracellular oxidative state at tissue-achievable doses, according to Posadino et al. It leads to mitochondrial damage and endothelial cell death (Posadino et al., 2015). Finally, the researchers concluded RES affected endothelial cells biphasically depending on the concentration. *In vitro*, RES at low concentrations reduced the oxidative state of endothelial cells. RES (≥10 µM) increased the status of oxidative stress at higher concentrations *in vitro* (Posadino et al., 2015). Overall, the adverse effects associated with RES use correlate with drug dose. Compared with the overwhelming benefits of RES, its side effects appear mild and sporadic.

Currently, there are many clinical cases that support the use of RES in the prevention and treatment of nervous system disorders. In patients with acute ischemic stroke, rt-PA is one of the most successful treatment options. However, their narrow therapeutic window limits their therapeutic benefits (Lansberg et al., 2012). As is reported, co-administration of RES and rt-PA could extend the time-bound therapeutic window in cerebral stroke patients (Chen et al., 2016). Additionally, they also suggest a potential role for the combination of RES and rt-PA treatment to improve BBB integrity. There has also been researching on the effects of RES on neurological and cognitive disorders in overweight but otherwise healthy individuals as well. Memory performance was improved by RES by increasing hippocampal functional connectivity and improving glucose metabolism (Witte et al., 2014). Interestingly, it was found that RES altered cerebral blood flow in healthy adults. During this study, a dose-dependent increase in cerebral blood flow was observed following two doses of trans-resveratrol (250 and 500 mg) (Kennedy et al., 2010). While RES has been reported to be beneficial for a number of human diseases, it is currently not prescribed for them. Many clinical trials still ongoing. As knowledge of the health benefits of RES increases, the clinical utility is likely to become more apparent and widely accepted.

## Conclusion and future direction

CVD severely impacts the patient’s quality of life. Treatments of cerebrovascular disorders have developed significantly, such as intravenous (IV) tissue plasminogen activator (IV-tPA), intra-arterial therapy (IAT) for ischemic stroke, and endovascular embolization of vascular malformations. However, thrombolysis has a relatively narrow therapeutic time window (4.5 h) and the risk of severe adverse effects such as hemorrhagic transformation. These treatments are effective, but it is limited to use in a small proportion of patients. Therefore, developing new therapeutic agents are necessary for the treatment of CVD.

Natural products and their derivatives are widely used as treatments for many diseases. RES has various properties like low cytotoxicity, low cost, and extensive sources. More importantly, oxidative stress also becomes a prominent target of RES. Taken together, RES has therapeutic value in CVD mainly by inhibiting excessive ROS production and elevating antioxidant enzyme activity. Despite that, RES is limitedly used because of its low aqueous solubility and bioavailability. To overcome these defects, cyclodextrins and nanoparticles are developed as drug carriers, which can be applied for enhancement of the solubility, stability, and absorption rate. In addition, an effective sustained-release drug delivery system may decrease the early degradation in the intestine and liver. These innovative approaches have significantly improved the pharmacokinetics of RES, providing a promising treatment for CVD.

## Glossary

Al: Aluminum
AMPK: Adenosine 5′-monophosphate (AMP)-activated protein kinase
APE1: Apurinic/apyrimidinic endonuclease one
AQP4: Aquaporin four
ARE: Antioxidant response element
ATP: Adenosine triphosphate
BBB: Blood-brain barrier disruption
BCCAO: Bilateral common carotid artery occlusion
CaMKIV: Calmodulin kinase IV
cAMP: Cyclic adenosine monophosphate
CAT: Catalase
CCH: Chronic cerebral hypoperfusion
CoA: CoenzymeA
COX2: Cyclo-oxygenase two
CREB: cAMP-response element binding protein
Cu: Copper
CVD: Cerebrovascular diseases
DCX: Doublecortin
DNA: Deoxyribonucleic acid
eNOS: Endothelial nitric oxide synthase
ER: Endoplasmic reticulum
Fe: Ferrum
GPx: Glutathione peroxidase
GFAP: Glial fibrillary acidic protein
GLUT3: Glucose transporter3
GRP78: Glucose-regulated protein 78
GSH: Glutathione
GSH-Pxs: Glutathione peroxidase
GST: Glutathione-s-transferase
H_2_O_2_: Hydrogen peroxide
HO-1: Heme oxygenase-1
I/R: Ischemic reperfusion
IL-1β: Interleukin-1β
JAK: Janus kinase
Keap1: Kelch-like ECH- associated protein l
LDH: Lactate dehydrogenase
MCAO/R: Middle cerebral artery occlusion/reperfusion
MDA: Malondialdehyde
Mg: Magnesium
MMP: Matrix metalloproteinase
MnSOD: Manganese superoxide dismutase
MPO: Myeloperoxidase
MPTP: Mitochondrial permeability transition pore
mTOR: Mechanistic target of rapamycin
mtDNA: Mitochondrial DNA
NF-κB: Nuclear factor kappa-B
NGF: Nerve growth factor
NLRP3: NOD-like receptor protein three inflammasome
NMDA: National Marine Distributors Association
NO: Nitric oxide
NQO1: NAD(P)H qui-none oxidoreductase one
NRF-1: Respiratory factors 1
Nrf2: NF-E2-related factor 2
OGD/R: Oxygen-glucose deprivation/reperfusion
PGC-1α: Peroxisome proliferator-activated receptor-gamma coactivator (PGC)-1alpha
PI3K/Akt: Phosphatidylinositol three kinase/Protein Kinase B
PINK1: PTEN-induced putative kinase protein one
PKA: Protein kinase A
PSA-NCAM: Polysialylated-neural cell adhesion molecule
PTEN: Phosphatase and tensin homolog deleted on chromosome ten
RCCA: Right common carotid artery
RES: Resveratrol
RMCA: Right Middle Cerebral Artery
ROS: Reactive oxygen species
RPC: RES preconditioning
rt-PA: Recombinant tissue plasminogen activator
SAH: Subarachnoid hemorrhage
Se: Selenium
SIRT1: Sirtuin1
SOD: Superoxide dismutase
STAT3: Signal transducer and activator of transcription3
SUR1: Sulfonylurea Receptor1
TAC: tricarboxylic acid cycle
TFAM: Transcription factor A mitochondrial
TGF-β-ERK: Transforming growth factor-β-Extracellular-signal-regulated kinases
TIMP-1: Tissue inhibitor of matrix metalloproteinases
TLR4: Toll-like receptor four
TNF-α: Tumor necrosis factor-α
TRPC6-MEK: Transient receptor potential channel 6/methyl ethyl ketone
VaD: Vascular dementia
VO: Vessel occlusion
Zn: Zinc
