# Supplementary material for: Antioxidant and neuroprotective actions of resveratrol in cerebrovascular diseases
Source: Front Pharmacol. 2022 Sep 5;13:948889. doi: 10.3389/fphar.2022.948889 (PMC9483202; doi:10.3389/fphar.2022.948889)
Supplement: Supplementary file 1 [file Table1.DOCX]

Table1. Summary of the most relevant preclinical studies evaluating the effects of RES administration to animals subjected to CVD including ischemic stroke, haemorrhage stroke and vascular dementia.

| **Disease** | **Model** | **Dose and duration of study** | **Outcome of study** | **Reference** |
| --- | --- | --- | --- | --- |
| Ischemic stroke | Middle cerebral artery occlusion/reperfusion (MCAO/R), SD rats;  Oxygen-glucose deprivation/reperfusion(OGD/R), Primary cortical neurons | RES30mg/kg, intraperitoneally, for 7 days  RES 5μmol/L, for 24h | RES reduced neurological deficit scores, promoted proliferation of neural stem cell，inhibited astrocyte and microglia activation by the Shh signaling pathway. | (Yu et al. 2021) |
|  | Right common carotid artery (RCCA), SD rats | RES 20mg/kg, intraperitoneally, for 10days | RES reduced levels of MDA, Ferrum (Fe), Copper(Cu), Aluminum(Al) and increased the anti-oxidants enzyme SOD and CAT activity. | (Lin et al. 2021) |
|  | OGD/R, Primary cortical neurons | RES 10µM, prior to reoxygenation. | RES improved cell viability, suppressed apoptosis and oxidative stress by stimulating the PTEN-induced putative kinase protein 1(PINK1)/Parkin‑mediated pathway. | (Ye, Wu, and Li 2021) |
|  | Right Middle Cerebral Artery (RMCA), SD rats | RES20mg/kg, intraperitoneally, for 10 days | RES increased trace element concentrations of Magnesium(Mg), Zinc(Zn), Selenium(Se), SOD and CAT antioxidant activity. | (Ro, Liu, and Lin 2021) |
|  | MCAO/R，Wistar rats | RES1.9mg/kg, tail vein injection, at the onset of reperfusion | RES reduced the cerebral region damage and diminishes glucose transporter 3 (GLUT3) expression at the mRNA and protein level in astrocytes which might depend on adenosine 5‘-monophosphate (AMP)-activated protein kinase(AMPK) activation. | (Gutiérrez Aguilar et al. 2020) |
|  | MCAO, SD rats | RES 30 mg/kg, intraperitoneally | RES improved the neurological behavior，brain edema and brain infraction by upregulating the p-Akt and p-glycogensynthasekinase-3β (p-GSK-3β) expression levels. | (Park et al. 2019) |
|  | MCAO, Wistar rats;  Excitotoxicity, Primary cortical neuronal | RES1.8 mg/kg, tail vein injection  RES 40μM, after excitotoxicity | RES decreased the infarct area，the production of superoxide anion, the overload of intracellular Ca^2+^ and increased the levels of phosphorylated AMPK | (Pineda-Ramírez et al. 2020) |
|  | OGD, SH-SY5Y cells | RES 10 μM, for 48 h after OGD | RES rescued mitochondrial deficiency, increased the Bcl-2 and CREB expression, inhibited caspase 3 and 9 activity via increasing expression of AMPK and p-AMPK | (Lin et al. 2020) |
|  | MCAO/R, SD rats | RES 10,100 mg/kg, intraperitoneally at 2 h  On the onset of ischemia. | RES significantly reduced neurological deficit, cerebral infarct sizes, neuronal injury, and decreased inflammation, BBB disruption by downregulation of the toll-like receptor 4(TLR4) pathway. | (Lei et al. 2019) |
|  | MCAO, C57BL/6 mice | RES 200 mg/kg, intraperitoneally for 3 days | RES attenuated systemic infammation and neuroinfammation by modulating intestinal fora-mediated Th17/Tregs and Th1/Th2 polarity shift in SI-LP. | (Dou et al. 2019) |
|  | MCAO，SD rats | RES 30 mg/kg, intraperitoneally, for 7 days | RES significantly decreased neuronal damage, attenuated neuronal apoptosis via upregulating the PI3K/AKT/mTOR pathway by activating janus kinase 2(JAK2)/signal transducer and activator of transcription3(STAT3). | (Hou et al. 2018) |
|  | MCAO, SD rats | RES 100 mg/kg, intraperitoneally at 2 and 12 h after the onset of ischemia | RES significantly reduced the enzymatic activity of myeloperoxidase (MPO)，suppressed the inflammatory factors，and upregulated the expression of cyclo-oxygen-ase 2(COX2) by activating PI3K/Akt pathway. | (Lei and Chen 2018) |
|  | OGD/R, Primary rat cortical neurons | RES 40 mmol/L, for 24 h before OGD/R | RES treatment at different times increased neuronal viability, decreased the lactate dehydrogenase(LDH) and SOD activity, and inhibited neuronal apoptosis via enhancing the activation of the Nrf2 pathway. | (Yang et al. 2018) |
|  | MCAO/R, SD rats | RES 20 mg/kg, intraperitoneally for 7 days. | RES alleviated cognitive impairment, downregulated inflammatory cytokines via modulating JAK/ERK/STAT pathway. | (Chang et al. 2018) |
|  | OGD/R, HT22 cell | RES 10µM | RES attenuated cytotoxicity, oxidative stress and repair deoxyribonucleic acid (DNA) damage by upregulating apurinic/apyrimidinic endonuclease 1(APE1) activity and level. | (Jia et al. 2017) |
|  | MCAO, SD rats | RES 100 mg/kg, intraperitoneally at onset of reperfusion | RES attenuated inflammation, upregulated autophagy by inhibiting NOD-like receptor protein 3 inflammasome (NLRP3) inflammasome activation through Sirt1-dependent autophagy activity. | (He et al. 2017) |
|  | 2-Vessel occlusion(VO), Wistar rats | RES 20 mg/kg, intraperitoneally, for30 days | RES exerted cerebral protection and inhibited inflammation by reducing interleukin-1β (IL-1β) and upregulating osteopontin. | (Al Dera 2017) |
|  | MCAO, Wistar rats | RES 20mg/kg, orally, for 30 days | RES pre-administration reduced oxidative stress, inflammation, apoptosis, enhanced the levels of oxidized forms of DJ-1, and increased the Nrf2 expression via PI3K/Akt pathway activation. | (Abdel-Aleem et al. 2016) |
|  | MCAO, SD rats | RES 50 mg/kg, intraperitoneally, for 7 days | RES increased levels of IL-10, decreased tumor necrosis factor-α (TNF-α) and IL-6, increased frequencies of Tregs in the spleens and ischemic hemisphere, and improved the frequency and suppressive function of Tregs in the spleens. | (Yang et al. 2016) |
|  | MCAO, SD rats | RES 30 mg/kg, intraperitoneally at 1, 4, 6, 12, or 24 h before ischemia. | RES before ischemia exerts a potent neuroprotective effect with an efficacious time-window of at least 4 h via the national marine distributors association (NMDA) receptor-mediated ERK1/2-cAMP-response element binding protein (CREB) pathway . | (Li, Fang, et al. 2016) |
|  | MCAO, SD rats | RES 20 mg/kg, intraperitoneally, for 5 days | RES significantly reduced adenosine triphosphate (ATP) energy consumption and exerted neuroprotection by inhibiting PDEs and regulating the cyclic adenosine monophosphate(cAMP)/AMPK/SIRT1 pathway. | (Wan et al. 2016) |
|  | MCAO, SD rats | RES 20 mg/kg, intraperitoneally at 0 and 20 h following reperfusion. | RES prevented against brain injury through ameliorating oxidative stress and reducing AQP4 expression. | (Ma et al. 2013) |
|  | 4-VO，Wistar rats | RES(1,10mg/kg), intraperitoneally, for 21days | RES attenuates doublecortin(DCX)/polysialylated-neural cell adhesion molecule(PSA-NCAM) expression, increased angiogenesis, improved spatial memory retention, and regulated corticosterone secretion. | (Girbovan et al. 2016) |
|  | 4-VO，Wistar rats | RES(1,10mg/kg), intraperitoneally, for 21days. | RES exerted brain protection by increasing GLT-1 expression and inhibiting CD11b/c and glial fibrillary acidic protein (GFAP) expression. | (Girbovan and Plamondon 2015) |
|  | MCAO, SD rats | RES 50 mg/kg, intraperitoneally | RES attenuated the cerebral ischemia by maintaining the integrity of BBB via regulation of MMP-9 and tissue inhibitor of matrix metalloproteinases-1 (TIMP-1). | (Wei et al. 2015) |
|  | MCAO, SD rats | RES (0.1 and 1μM), Intracortical injection | RES exerted neuroprotection by activating either estrogen receptor subtype within the ischemic cortex of rats. | (Saleh, Connell, and Saleh 2013) |
|  | MCAO, SD rats | RES 200 mg/kg, intraperitoneally, for 6 days | RES protected the brain through the Transient receptor potential channel 6/Methyl ethyl ketone(TRPC6-MEK)-CREB and TRPC6-CaMKIV-CREB pathways. | (Lin et al. 2013) |
| Cerebral hemorrhage | Ischemic reperfusion(I/R), SD rats | RES 10 mg/kg, intravenously, for 20 days | RES ameliorated brain injury and attenuated neuronal apoptosis by downregulating the TGF-β-ERK pathway. | (Zhao et al. 2019) |
|  | SAH, SD rats | RES 100 mg/kg, intraperitoneally, 48 h prior to SAH | RES exerted neuroprotective effects, prevented BBB disruption through the SIRT1/p53 signal pathway. | (Qian et al. 2017) |
|  | SAH, SD rats | RES 60 mg/kg，intraperitoneally, 2 and 12 h post SAH | RES provided neuroprotection, inhibited mitochondrial-dependent apoptosis, improved mitochondrial biogenesis and antioxidative ability by activating the PGC-1α signaling pathway. | (Zhou et al. 2021) |
|  | SAH, SD rats | RES (20、60) mg/kg, intraperitoneally， at 2 and 24 h after initial bleeding | RES attenuated neuronal apoptosis by the PI3K/Akt signaling. | (Zhou et al. 2014) |
|  | SAH, SD rats | RES 30 mL/kg, intraperitoneally, at 6 h after SAH | RES promoted brain functional recovery, prevented BBB disruption, inhibited the activation of nuclear factor kappa-B(NF-κB) and downregulation of MMP-9 expression. | (Shao et al. 2014) |
| Vascular dementia | Chronic cerebral hypoperfusion(CCH)，Wistar rats | RES 40 mg/kg,intraperitoneally, for 4 weeks | RES effectively restore the synaptic plasticity and improved spatial memory via PKA-CREB activation. | (Li, Wang, et al. 2016) |
|  | 2-VO, Wistar rats | RES 20 mg/kg，intraperitoneally, for 7 days | RES attenuated pyramidal cell death in the CA1 hippocampal subfield, prevented both spatial working and reference memory impairments, increased the nerve growth factor (NGF) levels. | (Anastácio et al. 2014) |
|  | Bilateral common carotid artery occlusion (BCCAO), SD rats | RES 20 ml/kg, intraperitoneally , for 4 weeks | RES exhibited neuroprotective effects, inhibited the apoptosis and oxidative stress injury. | (Zhang et al. 2019) |
|  | BCCAO, SD rats | RES 50 mg/kg, intragastrically, for 9 weeks: | RES improved cognitive function, reduced neuronal damage and neuronal apoptosis by activating autophagy and regulating the Akt/mTOR signaling pathway. | (Wang et al. 2019) |
